# Tuberculosis of the radius in a child

**DOI:** 10.1590/0100-3984.2017.0114

**Published:** 2019

**Authors:** Vanessa Maria Terra Gomes, Teresa Cristina Sarmet dos Santos, Luis Alcides Quevedo Cañete, Caroline Figueira, Rebeca Albuquerque

**Affiliations:** 1 Complexo Hospitalar de Niterói (CHN), Niterói, RJ, Brazil.

**Figure 1:**
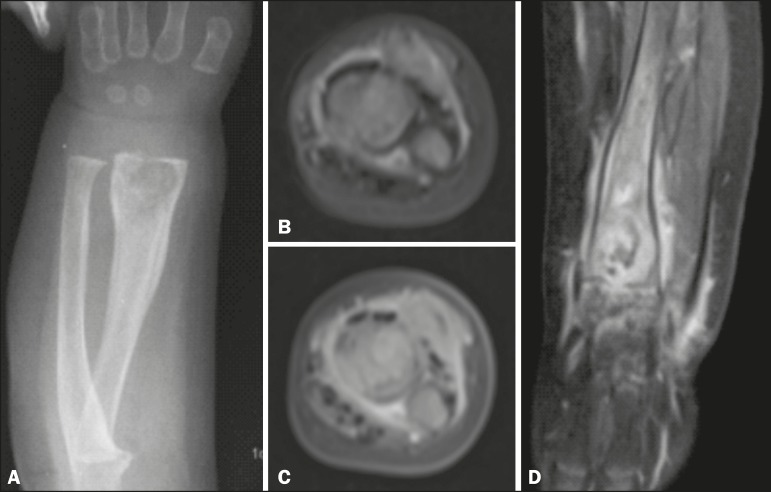
**A:** Anteroposterior X-ray of the forearm. Round osteolytic formation with partially defined margins, cortical irregularity, and periosteal reaction in the distal third of the radius. **B:** Axial proton density-weighted MRI. Expansile ill-defined solid heterogeneous lesion in the bone marrow of the distal metaphysis of the radius. Note the linear image with a hyperintense signal in the metadiaphysis and cortical discontinuity suggestive of fracture. **C:** Contrast-enhanced axial T1-weighted MRI with fat suppression. The signal intensity is similar to that of cartilaginous tissue, with hyperintense foci. **D:** Contrast-enhanced coronal T1- weighted MRI with fat suppression. Note that the lesion focally extends beyond the physis and infiltrates the perilesional soft tissue, with significant gadolinium enhancement, persistence of small loculated lesions with hypointense signals, and fluid infiltration, as well as enhancement of the joint spaces, muscle, and subcutaneous tissue.

Dear Editor,

A 9-month-old male infant was admitted to the emergency room after trauma to the left wrist. An X-ray of the forearm showed fracture of the distal radius. The limb was immobilized, and the patient was referred for outpatient follow-up. One month later, the patient presented with weight loss and bulging of the region after early removal of immobilization. On physical examination, the distal third of the left forearm presented edema and tenderness, with no joint locking of the wrist. The patient underwent another X-ray (Figure 1A) and a magnetic resonance imaging (MRI) scan (Figures 1B, 1C and 1D), followed by immobilization of the forearm with a sugar-tong splint and administration of oral analgesics. The patient was again referred for outpatient follow-up. The pathology study was conclusive for bone tuberculosis, and the patient was started on a therapeutic regimen.

Two billion people are currently infected with *Mycobacterium tuberculosis*, and 8-9 million of those people have or will develop active tuberculosis^(^1^)^. Tuberculosis is a significant health problem in low- and middle-income countries. In 2012, there were 71,230 new cases of tuberculosis reported in Brazil, with an incidence rate of 36.7/100,000 population for all forms of the disease^(^1^-^3^)^. In that same year, in the state of Rio de Janeiro alone, 10,871 new cases were reported^(^1^)^.

After entering the body through the airways, *M. tuberculosis* can disseminate to any organ, especially if there is weakening of the immune response^(^4^)^. Diagnosing the extrapulmonary forms of the disease is more difficult due to the location of the lesions and because they are paucibacillary, bacteriological confirmation being obtained in only approximately one fourth of the cases. Imaging findings are usually nonspecific^(^4^)^.

Bone tuberculosis is an uncommon disease, affecting 10-15% of all patients with tuberculosis^(^5^-^7^)^. Bone and joint involvement is more common in pediatric and elderly patients. Although such involvement is usually secondary to hematogenous dissemination, it may also occur through lymphatic or contiguous spread^(^4^,^^5^^)^.

Tuberculosis can affect the entire skeleton. The most common site is the spine, whereas the radius is rarely affected. A common clinical manifestation of bone tuberculosis is monoarticular lesion, trauma involving the affected joint often being reported. Radiographic findings include osteolytic lesions with irregular borders, surrounded by areas of sclerosis. Findings of bone lesions with cystic cavities on X-rays are nonspecific because they mimic pyogenic osteomyelitis, fungal infection, metastasis, telangiectatic osteosarcoma, aneurysmal cyst, sarcoidosis, eosinophilic granuloma, and chordoma^(^8^-^10^)^. Establishing a diagnosis of bone tuberculosis is difficult mainly because of the indolent nature and nonspecific findings of the condition, which lead to an increase in morbidity and poorer prognoses^(^4^,^^6^^,^^11^^)^.

In conclusion, bone tuberculosis at uncommon sites is difficult to diagnose and can often be misdiagnosed as a tumor, because the clinical manifestations and imaging findings are similar. The physician should always bear in mind the possibility of *M. tuberculosis* infection, especially in areas endemic for the disease, and should be cautious in regard to the differential diagnoses, determining whether or not there is a need for biopsy, given that delayed treatment and overtreatment can both cause harm to the patient.
